# W. J. Hill, M.R.C.S., L.R.C.P.

**Published:** 1917-12

**Authors:** 


### Obituary.

### W. J. HILL.

Clevedon has lost a good friend and skilful practitioner by the death of Walter Hill on June 2nd, while to his former colleagues at the Bristol Royal Infirmary his untimely and unexpected death at the age of 53 makes a deeply-deplored gap in the alumni of the Bristol School, where he entered as a student in 1880, subsequently taking his M.R.C.S. and L.R.C.P. Remarkable for his quiet, almost retiring disposition, he nevertheless made many life-long friendships amongst his fellow-students, and in later life amongst both the well-to-do class and the poorer inhabitants of Clevedon. Since 1901, for sixteen years he had been the Medical Officer of Health for the Urban District of Clevedon, and he was also Medical Officer to the Dispensary and to the Clevedon Cottage Hospital for many years.

For several years his health, never robust, had suffered from the heavy strain of medical practice, but the additional burdens involved in his constant endeavour to make up for the shortage of medical practitioners in Clevedon and serve his confrères engaged on war work proved too great a strain, for latterly he worked very hard at the local Red Cross Hospital.

We recall the days when in 1886 he was House Physician at the Bristol Royal Infirmary, and the lovable, kindly, keen neophyte who never spared or thought of himself. He was just the type of man that wins the affections and regard of patients and of all those who come into his life. To Mrs. Hill, who, too, has many old friends at the Royal Infirmary, we offer sincere sympathy.

